# Putting Consumers at the Center in a Context of Limited Choice and Availability of Modern Contraception in Luanda, Angola. Authors' Response to “Assessing Angola's Contraceptive Market Landscape”

**DOI:** 10.9745/GHSP-D-17-00295

**Published:** 2017-09-27

**Authors:** Benjamin Nieto-Andrade, Eva Fidel, Rebecca Simmons, Dana Sievers, Anya Fedorova, Suzanne Bell, Karen Weidert, Ndola Prata

**Affiliations:** a Population Services International/Angola, Luanda, Angola.; b Institute for Reproductive Health, Georgetown University, Washington, DC, USA.; c Population Services International, Washington, DC, USA.; d Johns Hopkins Bloomberg School of Public Health, Baltimore, MD, USA.; e Bixby Center for Population, Health, and Sustainability, University of California, Berkeley, Berkeley, CA, USA.

See related articles by Nieto-Andrade et al. and by Harrison.

We welcome the opportunity to respond to Harrison's Letter to the Editor regarding our GHSP article “Women's Limited Choice and Availability of Modern Contraception at Retail Outlets and Public-Sector Facilities in Luanda, Angola, 2012–2015.” There is little recent market data available for Angola, and this article was an effort to share what we had learned about product availability in retail outlets and public-sector facilities in Luanda. We welcome debate and hope that the gaps identified in the market will inspire others to act.

Harrison makes 3 main points in her Letter to the Editor, and here we respond to each in turn.

## ANGOLA'S ECONOMIC CRISIS

We agree with Harrison that the economic crisis, inflation, and shortage of foreign exchange are contributing factors affecting supply and availability, a point that our original article discusses as well. As supply constricts, increase in price is a common outcome.

## MEASURING MARKET COMPETITIVENESS

Harrison questions our statement that there is limited choice and availability of contraceptives in Angola. Instead, she seems to suggest that contraceptive prevalence drives the current availability of modern contraceptive methods and that this relationship is unidirectional. We contend that the relationship between the modern contraceptive prevalence rate (mCPR) and availability of contraceptive methods is circular. We believe one of the main reasons mCPR is low in Angola is because contraceptive products and services are not easily available. A recent analysis of international data from 1982 to 2009 found that for each additional method available to at least half of the population, the percentage of married women using a modern method increases by 4 to 8 percentage points.^1^

We believe one of the main reasons mCPR is low in Angola is because contraceptive products and services are not easily available.

We agree with Harrison that along with the number of brands, the number of manufacturers or distributors in a market is also worth considering. Brands do “speak” to consumer segments and offer different price points, thereby increasing the likelihood that a consumer finds a choice that's right for her. For that reason, the number of brands remains a useful, but not the sole, measure of choice in a market.

In our original article, we state that public health policies must ensure the availability and affordability of contraceptives on the market and expand the range of options for women. Harrison argues “… that public health policies should instead support fair market competition and optimize the use of both public and private resources.” On that point, we agree with Harrison on the need to optimize use of public and private health resources, but we also believe that when public health goals are paramount, policy must consider public health outcomes in addition to the goal of creating a competitive market place.

We agree that subsidies need to be discrete and targeted to market failures in which market players are likely to underinvest. It is one of the reasons why Population Services International (PSI) spends a disproportionate amount of funding on health behavior change. If such investments were made by the commercial sector, they would need to be recouped from consumers, making health products and services unaffordable for most. Using subsidies and/or government policies to correct market failures could help to stimulate both demand and interest in market entry by commercial actors.

Using subsidies and/or government policies to correct market failures could help to stimulate both demand and interest in market entry by commercial actors.

The questions on timing of phasing out subsidy and its effect in the market, although not a topic of our original article, are very interesting and should be explored further. Nevertheless, it is worth noting that PSI no longer uses donor subsidy to market its condom brands in Angola, as these products have become fully sustainable.

## CLARITY ON PSI'S OWN INVOLVEMENT IN THE MARKET

PSI seeks to put the consumer at the center and bring health care closer to her. In contraceptive markets, this means ensuring women can easily access a broad range of contraceptive choices that are directly available on the market. In the oral contraceptive market in Angola, where PSI has not been playing a role until recently, there is a paucity of third-generation pills that are more suited to new and young users. With seed money from the Swedish government (the Swedish International Development Cooperation Agency, or Sida), in May 2017 PSI launched a third-generation oral contraceptive pill (note: our original article was accepted in November 2016) and will soon launch an emergency contraceptive, with the aim of providing additional choices to young women in Angola. With these oral contraceptive products, PSI is advancing a cost-recovery Social Enterprise model that will not require continuous subsidy from external sources.

On leakage, PSI believes in the role of public-sector subsidies to provide free contraceptives to consumers who cannot afford them. That subsidy is wasted when products meant for free distribution are misappropriated, essentially providing a subsidized product to consumers with ability to pay, and enriching those who manipulate the supply chain. Having found evidence of this in the marketplace in Luanda, the authors felt it important to share the finding.

Again, we offer our thanks to Harrison and to GHSP for this opportunity to discuss the contraceptive market in Angola.

## Notes

**Competing Interests:** Dr. Nieto-Andrade reports that PSI (www.psi.org), the authors' organization, is known as a nonprofit organization that has been distributing family planning products around the globe (among other products and services) for decades. Its goal is to improve the health and well-being of populations through social marketing and behavior change communication. PSI had received a grant from the U.S. Agency for International Development (USAID) to implement the “Ouakula” project in Angola in the areas of HIV, malaria, and family planning (2011–2016). The research studies to which Harrison's letter refers were funded by this USAID grant as part of program monitoring. PSI strongly values evidence-based decision making and uses data, as presented in our original GHSP article and in this letter, to design strategies that improve people's health while respecting the role of other players in the market. The authors did not receive any payment from any source to write the present response letter or the original article that received comments from Harrison.
